# The value of microRNA-21 as a biomarker for the prognosis of lung cancer: A protocol for systematic review and meta-analysis

**DOI:** 10.1097/MD.0000000000021483

**Published:** 2020-08-14

**Authors:** Wei Zhang, Lin Wei, Rong Luo, Hui Liu, Jing Chen

**Affiliations:** a Department of Medical Oncology, Sichuan Cancer Hospital & Institute/Sichuan Cancer Center/School of Medicine,University of Electronic Science and Technology of China; b Department of Medical Oncology, West China fourth hospital of Sichuan University, chendu, P. R. China.

**Keywords:** lung cancer, meta-analysis, miRNA-21, prognosis

## Abstract

**Backgroud:**

More and more studies are investigating the influence of the expression of MicroRNA-21 (miRNA-21) on prognosis and clinical significance in patients with lung cancer, but the results are contradictory and uncertain. A meta-analysis was conducted with controversial data to accurately assess the issue.

**Methods:**

A detailed search of relevant research in Wanfang, Chinese Biomedical Literature Database, Chinese National Knowledge Infrastructure (CNKI), the Chongqing VIP Chinese Science and Technology Periodical Database, PubMed, Embase, Web of Science and other databases. Two reviewers independently conducted data extraction and literature quality evaluation. Odd ratio and its 95% confidence intervals were used to evaluate the relationship between miRNA-21 and clinicopathological characteristics of lung cancer patients. Hazard ratios and its 95% confidence intervals To assess the prognostic effect of miRNA-21 on overall survival and disease-free survival. Meta analysis was performed using RevMan 5.3 and Stata 14.0 software.

**Results:**

This study will provide a high-quality evidence-based medical evidence of the correlations between miRNA-21 expression and overall survival, disease-free survival and clinicopathological features.

**Conclusion:**

The study will provide updated evidence to evaluate whether the expression of miRNA-21 is in association with poor prognosis in patients with lung cancer.

**Ethics and dissemination:**

The private information from individuals will not publish. This systematic review also will not involve endangering participant rights. Ethical approval is not available. The results may be published in a peer- reviewed journal or disseminated in relevant conferences.

**OSF REGISTRATION NUMBER:**

DOI 10.17605/OSF.IO/X3MD6

## 1 Introduction

It is estimated that there will be 1735350 new cases of cancer patients and 609640 deaths, according to the 2018 National Cancer Center statistics.^[1]^ This shows that cancer is still the main public health problem and challenge we face. Lung cancer is 1 of the malignant tumors with high morbidity and mortality worldwide and its prognosis is poor, the 5-year survival rate is <15%.^[2]^ According to the CONCORD-3 project of the American Cancer Society and the Centers for Disease Control and Prevention, the global lung cancer incidence accounts for about 13% of all cancer incidences, of which 12.5% in developed regions and 13.3% in underdeveloped regions.^[3]^ In 2018, the number of new lung cancer deaths and deaths in the world was 2.094 million and 1.76 million respectively, and the number of new lung cancer deaths and deaths in the Chinese population was 774,000 and 691,000, respectively. The overall morbidity and mortality are increasing year by year.^[3]^

The onset of lung cancer is often hidden. Most patients are diagnosed in the middle and late stages and have lost the opportunity for radical treatment, so the main treatment measures at this stage are chemotherapy or targeted therapy. But Many patients have poor prognosis due to the presence of cancer cell metastasis and insensitivity to radiotherapy and chemotherapy, and the mortality rate is as high as 85%.^[4]^ Tumor marker refers to a type of substance that exists or is secreted in tumor cells and can reflect tumor occurrence and development, and monitor tumor response to treatment, including tumor antigens, hormones, glycoproteins, enzymes and isoenzymes, oncogenes, and tumor suppressor gene.^[5]^ Therefore, it is of great significance to actively search for and study factors related to the prognosis of lung cancer.

MicroRNA (miRNA) is a type of small RNA with a length of 18∼25 amino acids and no protein coding function.^[6]^ miRNA can bind with the 3’UTR sequence of mRNA to degrade mRNA or inhibit the transcription of mRNA, thereby participating in the biological processes of regulating cell proliferation, differentiation, apoptosis and innate immunity.^[7]^ Gallach and so on have reported that miRNA may be involved as an oncogene or tumor suppressor gene in the occurrence and development of various tumors including lung cancer.^[8]^ Multiple studies have shown that the high expression of miRNA-21 is closely related to the survival of lung cancer patients, but there are also large differences in the research results. Some studies have shown that the expression of miRNA-21 is positively correlated with the survival rate of lung cancer patients,^[9–13]^ but some studies have shown that the expression of miRNA-21 is negatively correlated with the survival rate of lung cancer patients.^[14,15]^ In order to more accurately analyze the effect of high expression of miRNA-21 on the survival of lung cancer patients. This study comprehensively searched the literature related to the expression of miRNA-21 and the prognosis of lung cancer patients, and used meta-analysis to evaluate the effect of high expression of miRNA-21 on the prognosis of lung cancer patients.

## 2 Methods

### 2.1 Study registration

This meta-analysis protocol is based on the Preferred Reporting Items for Systematic Reviews and meta-analysis Protocols (PRISMA-P) statement guidelines.^[1]^ The PRISMA-P checklist for the protocol is provided in the PRISMAP-checklist.

The protocol of the systematic review has been registered on Open Science Framework. The registration number is DOI 10.17605/OSF.IO/X3MD6

### 2.2 Data sources and search strategy

Wanfang, Chinese Biomedical Literature Database, Chinese National Knowledge Infrastructure, the Chongqing VIP Chinese Science and Technology Periodical Database, PubMed, Embase, Web of Science and other databases will be our electronic databases for retrieval. The retrieval time is from their inception to May 2020. The retrieval strategy will be created based on discussion of all the researchers on the basis of the Cochrane handbook guidelines. The following search terms will be used: “lung cancer”, “lung neoplasm”, “miRNA-21”, “microRNA-21”, “miR-21”, “prognostic”, and “survival”. The search strategy for PubMed is shown in Table 1. The retrieval strategy can be modified according to the actual situation of other electronic databases.

**Table 1:**
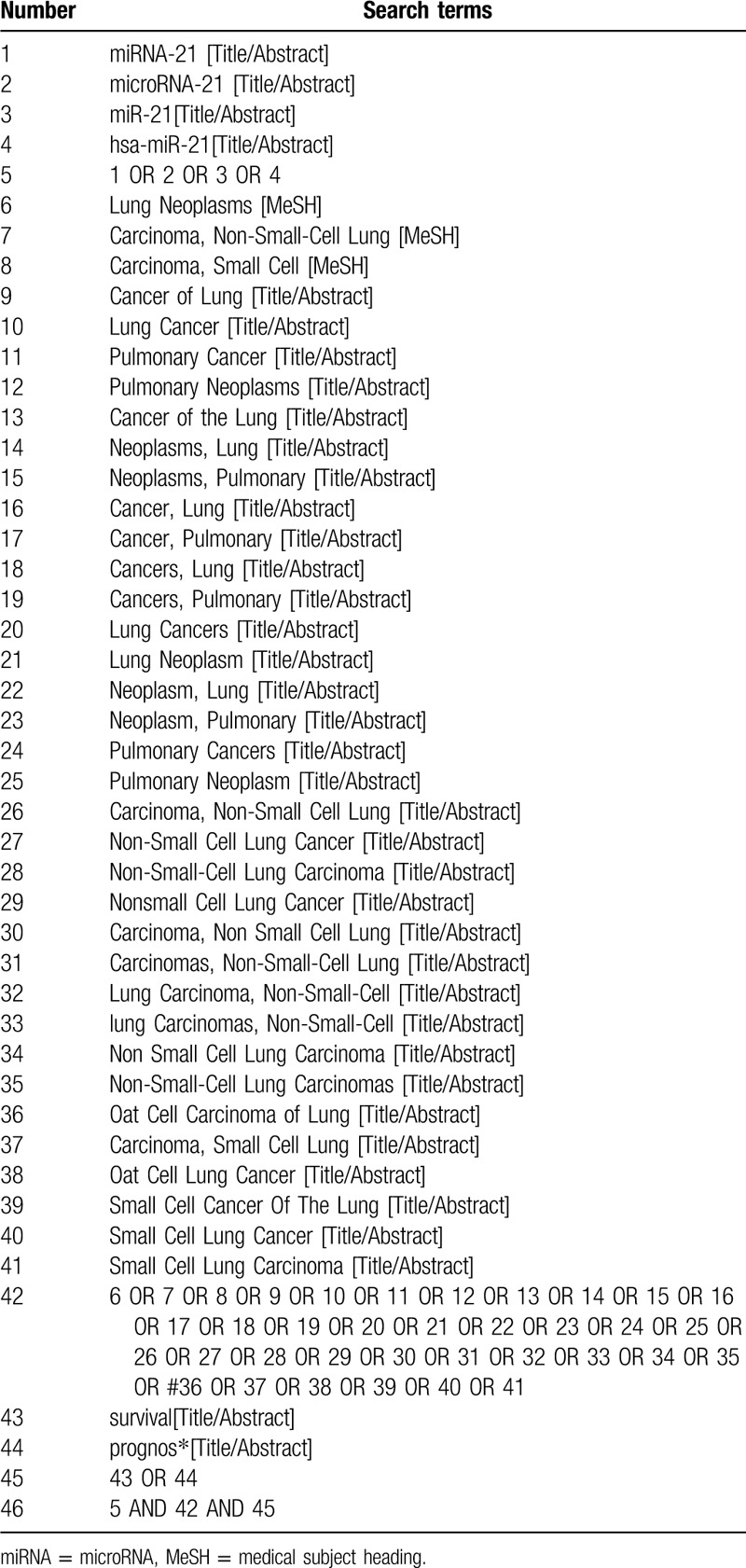
Search strategy in PubMed database. Table 1 PubMed search strategy.

### 2.3 Inclusion criteria for study selection

The included articles must meet the following inclusion criteria:

(1) Patients diagnosed with lung cancer based on pathology and histology.
(2) miRNA-21 expression in tumor tissues.
(3) Reported miRNA-21 survival-related data, including overall survival (OS) And disease-free survival (DFS)
(4) Patients are divided into miRNA-21 positive (high) and miRNA-21 negative (low).
(5) The article provides the relationship between miRNA-21 expression and clinical pathological characteristics.
(6) Published as full-text articles, original Chinese and English Research papers.

If there are repeated articles, choose articles with higher quality and larger sample size. Conference abstract, case series report, letters, animal experiments, lack of measurement indicators and survival Research are not included

### 2.4 Data collection and analysis

#### 2.4.1 Selection of studies

**Figure 1:**
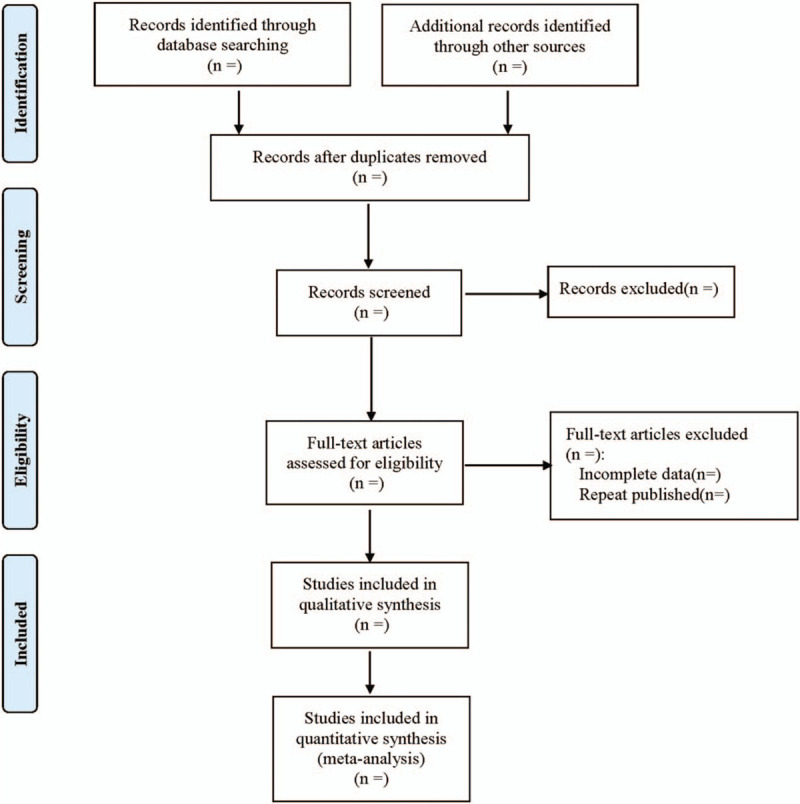
Flow diagram of study selection process.

In order to ensure the correctness of the included studies, all researchers have received evidence-based training, and adhered to the process summarized in the light of the PRISMA flow diagram (Fig. 1). All results retrieved from the electronic database are imported into endnote 8.0. First, delete duplicate documents through endnote 8.0. Then, the 2 researchers independently screened based on the title, abstract and keywords of the literature to remove irrelevant literature. The remaining literature will be further confirmed by the 2 researchers by reading the full text. Excluded studies and reasons for exclusion will be recorded. The differences between the 2 researchers will be resolved by consensus or by a third independent arbitrator.

#### 2.4.2 Data extraction and management

Data were double input to EpiData software (version 3.0; The EpiData Association, Odense, Denmark) by 2 investigators. Differences were resolved through group discussions. The information extracted includes the name of the first author and the year of publication, country, histology type, tumor stage, sample size, miRNA-21 detection method, and hazard ratios (HRs) and 95% confidence intervals (CIs) for OS, and DFS, if provided. We obtained HRs and 95% CIs from the Kaplan-Meier survival curves through using Engauge Digitizer version 4.1 (http://digitizer.sourceforge.net/).

### 2.5 Assessment of quality in included studies

Newcastle Ottawa Scale was used to evaluate the quality of the literature, including a total of 3 items and 8 items. The 2 researchers scored the literature according to the scored items, and the differences were resolved by group discussions. Selectivity from the study population The measurement of comparability, exposure between groups, and exposure factors were developed, with a perfect score of 9 points, and documents with a score of ≥7 were judged to be of higher quality

### 2.6 Measures of prognosis

OS and DFSwill be taken as prognostic outcomes. The results will be expressed as HRs with 95% CIs.

### 2.7 Management of missing data

If there is insufficient or missing data in the literature, we will contact the author via email to request the data. If the data is not available, we will only analyze the currently available data and discuss its potential impact.

### 2.8 Statistical analysis

Statistical analysis was performed using STATA 14.0 (STATA Corporation, College Station, TX) and RevMan 5.3 (The Nordic Cochrane Centre, The Cochrane Collaboration, 2014). The 95% CIs and HRs was used to evaluate the relationship between miRNA-21 expression and OS and DFS. Odds ratio and 95% CIs were used to evaluate the impact of miRNA-21 expression on clinicopathological characteristics. First, statistical heterogeneity tests were performed on the included studies.If there is no statistical heterogeneity among the included literatures (*I*^*2*^ ≤ 50%, *P* ≥ .1), a fixed effect model is used; when there is statistical heterogeneity among the included literatures (*P* < .1, *I*^*2*^ > 50%), the sources of heterogeneity will be analyzed. Clinical heterogeneity will be treated by subgroup analysis. In the absence of significant clinical heterogeneity and methodological heterogeneity, statistical heterogeneity will be considered, and random effects models will be used for analysis. If the clinical heterogeneity of the subgroup analysis is significantly higher, no meta-analysis will be performed, only a descriptive analysis.

### 2.9 Additional analysis

#### 2.9.1 Subgroup analysis

We will conduct a subgroup analysis based on the type of lung cancer, the detection method of miRNA-21 expression, the patient's age, race, and source of survival data.

#### 2.9.2 Sensitivity analysis

The sensitivity analysis of each index was carried out by 1-by-1 elimination method to check the stability of the results.

#### 2.9.3 Reporting bias

If the number of studies included in a certain outcome index is no less than 10, funnel chart is used to evaluate publication bias. Besides, Egger and Begg test were used for the evaluation of potential publication bias.

## 3 Discussion

Data from 36 cancer incidences and deaths in 185 countries around the world show that there were approximately 2.093 million (11.6%) new cases of lung cancer in the world, and approximately 17.61 million (18.4%) deaths, ranking first in all malignant tumors in 2018.^[1]^ lung cancer mortality rate is increasing year by year in China.^[17]^ Some studies have shown that the 5-year survival rate of early lung cancer can reach more than 90% after treatment, however, the 5-year survival rate after treatment of advanced lung cancer is less than 5%.^[18,19]^ Therefore, finding biomarkers with high specificity and high sensitivity has important clinical significance for the early diagnosis and prognosis of lung cancer.

In recent years, a large number of studies have shown that miRNA-21 plays an important role in the occurrence and development of lung cancer. Capodanno et al^[12]^ found that miRNA-21 is increased in lung cancer tissues, and miRNA-21 can be used as a biomarker to distinguish lung cancer tissues from normal tissues. Zhang et al^[20]^ found that the levels of miRNA-21 in the plasma of lung cancer patients were significantly higher than those of healthy people. In addition, the plasma miR-21 levels of lung cancer patients were significantly reduced after surgery. High expression of miRNA-21 is a risk factor for the prognosis of lung cancer.^[21]^ Liu et al^[22]^ found that the expression level of miRNA-21 in lung adenocarcinoma cells, squamous cell carcinoma cells and lung cancer tissues was significantly higher than that of human bronchial epithelial cells and adjacent tissues. miRNA-21 as an oncogene participates in regulating the occurrence and development of lung cancer by regulating the PTEN signaling pathway. Ma et al^[23]^ found that down-regulation of miRNA-21 can inhibit the proliferation of cells by inhibiting the PI3K/Akt signaling pathway, promote cell apoptosis, and enhance the sensitivity of radiation-resistant cells A549 to ionizing radiation. Yang et al^[24]^ found that miRNA-21 can be used as a biomarker for lung cancer. The AUC value of the ROC curve for the diagnosis of NSCLC is 0.81, and the sensitivity and specificity are 69% and 71%, respectively. Increased expression of miRNA-21 may be involved in the occurrence and development of lung cancer, resulting in decreased OS. To this end, we hope that this meta-analysis will provide more accurate and objective evidence for the relationship between miRNA-21 expression and prognosis in lung cancer patients.

Our research has certain limitations. First, the detection method and threshold of miRNA-21 expression may be different. In addition, there are various treatment methods for patients, such as surgery, chemotherapy, radiotherapy, targeted therapy, and immunotherapy. Therefore, there may be a risk of heterogeneity. In addition, this study only includes studies published in English and Chinese, so important studies or reports published in other languages may be omitted.

## Author contributions

Conceptualization: Wei Zhang and Jing Chen.

Data collection: Wei Zhang and Lin Wei.

Funding acquisition: Jing Chen.

Resources: Lin Wei and Rong Luo.

Software: Liu Hui.

Supervision: Lin Wei and Rong Luo.

Writing – original draft: Wei Zhang and Jing Chen.

Writing – review and editing: Wei Zhang and Jing Chen.
